# Optimal cutoff of the triglyceride to high-density lipoprotein cholesterol  ratio to detect cardiovascular risk factors among Han adults in Xinjiang

**DOI:** 10.1186/s41043-016-0067-8

**Published:** 2016-09-01

**Authors:** Hua-Yin Li, Bang-Dang Chen, Yi-Tong Ma, Yi-Ning Yang, Xiang Ma, Fen Liu, Zhen-Yan Fu, Xiang Xie, Xiao-Mei Li, Shuo Pan, Chun-Hui He, Ying-Ying Zheng, Yun Wu, Jing Tao, Chun-Lan Dong, Ting-Ting Wu

**Affiliations:** 1 Department of Cardiology, First Affiliated Hospital of Xinjiang Medical University, Urumqi, 830054 China; 2 Xinjiang Key Laboratory of Cardiovascular Disease, Clinical Medical Research Institute, First Affiliated Hospital of Xinjiang Medical University, Urumqi, China

**Keywords:** Cutoff, TG/HDL ratio, Cardiovascular risk factors, Han adults, Xinjiang

## Abstract

**Background:**

To determine whether TG/HDL-C ratio, which has been shown to be an indicator of the metabolic syndrome (MetS) and insulin resistance (IR), can predict cardiovascular risk factors in the Chinese Han population in Xinjiang.

**Methods:**

The cardiovascular risk survey (CRS) was conducted from October 2007 to March 2010. A total of 14,618 representative participants were selected using a four-stage stratified sampling method. A total of 5757 Han participants were included in the study. The present statistical analysis was restricted to the 5595 Han subjects who had complete anthropometric data. The sensitivity, specificity, and distance on the receiver operating characteristic (ROC) curve in each TG/HDL level were calculated. The shortest distance in the ROC curves was used to determine the optimal cutoff of the TG/HDL-C ratio for detecting cardiovascular risk factors.

**Results:**

The prevalence of hypertension, hypercholesterolemia, and hypertriglyceridemia was higher with higher TG/HDL-C ratio for both men and women. The TG/HDL-C ratio was positively associated with systolic blood pressure, diastolic blood pressure, and serum concentrations of total cholesterol. The optimal TG/HDL-C ratio cutoffs for predicting hypertension, dyslipidemia, diabetes, and ≥2 of these risk factors for Han adults in Xinjiang were 1.3, 1.3, 1.4, and 1.4 in men and 0.9, 1.0, 1.0, and 1.1 in women, respectively.

**Conclusions:**

The evaluation of TG/HDL-C ratio should be considered for one of cardiovascular risk factor predictors among Han adults in Xinjiang.

## Background

Cardiovascular disease (CVD) is the leading cause of death in the world, and mortality due to CVD is higher in low- and middle-income countries comparing with developed countries. It is expected to sharply increase the disease burden over the next 10 years [1]. CVD has become the leading cause of death in China [2]. It was estimated that 43.8 % of deaths in Chinese adults aged ≥40 years were attributed to heart disease and stroke during 1991–2000 in a national prospective cohort study [3]. Serum lipid levels were increasing in the Chinese population. Without effective intervention, atherosclerosis-related diseases may soar in the near future in China [4].

Lipid metabolic disorders have been proved as one of the pathogenesis of atherosclerosis, which is fundamental to the occurrence of CVD. Elevated serum lipid is one of the most important risk factors for cardiovascular disease in Western Countries as well as Asian populations [5–8]. High level of low-density lipoprotein cholesterol (LDL-C) is well established in the development of CHD, and the role of triglycerides (TG) and high-density lipoprotein cholesterol (HDL-C) remains controversial. Recently, some studies demonstrated that hypertriglyceridemia is an independent predictor of CHD and may be a stronger risk factor than previously thought to be [9–13]. The ratio of triglyceride/high-density lipoprotein cholesterol (TG/HDL-C), initially proposed by Gaziano et al., is an atherogenic index that has proven to be a highly significant independent predictor of cardiovascular disease [14]. Others have linked a high TG/HDL-C ratio to the risk of cardiovascular events [15, 16]. The cutoffs for the TG/HDL-C ratio are more often used to predict metabolic syndrome (MetS) and insulin resistance (IR) [17, 18], while few studies of CVD have focused on Han adults.

In Xinjiang Uighur Autonomous Region northwest of China, which is located in the center of Asia. The largest Chinese administrative division has a unique culture and lifestyle. The total Han population was 8.75 million in 2012 and takes 40.1 % of the total population in Xinjiang. Several studies have reported the condition of dyslipidemia among adults in Xinjiang [19–21], but the association between TG/HDL ratio and cardiovascular risk factors among Han adults in Xinjiang remains unknown. In this study, we aimed at to investigate the relationship between TG/HDL ratio and cardiovascular risk factors. We also tried to calculate the optimal cutoff points of TG/HDL ratio to predict cardiovascular risk factors among Han adults in Xinjiang.

## Methods

### Sample design

Eligible patients were selected from the cardiovascular risk survey (CRS) study, and the detailed description of the study population and the methods were described previously [22, 23]. Briefly, the CRS study used a 4-stage stratified sampling method to select a representative sample of the general population in Xinjiang, northwest of China. The research sites included Urumqi City, Kelamayi City, Fukang City, Turpan Prefecture, Hetian Prefecture, and Yili Prefecture. The time period was from October 2007 to March 2010. The selections were made from sampling units based on geographic area, sex, and age groups using household registries. The 4-stage stratified sampling method was as follows: Stage 1, according to population census data of Xinjiang in 2000, the areas mentioned above were selected based on population, ethnicity, geography, economic, and cultural development level, respectively. Stage 2, according to the ethnic aggregation status, one district or county was randomly selected from the Han population dominated area. Stage 3, one community or town (village) was randomly selected from each district or county. Stage 4, subjects aged above 35 years were randomly selected from each community or town (village) as research participants. The staff conducted surveys in households and administered questionnaires. The questionnaires included the demographic, socioeconomic, dietary, and medical history of each participant. In total, the CRS included 14,618 participants (5757 Hans, 4767 Uighurs, and 4094 Kazakhs). Five thousand five hundred ninety-five Han participants with complete data were enrolled in the present study. Two thousand seven hundred participants were male, and 2895 participants were female. The age of the participants were from 35 to 101 years old with the mean ± SD age of 53 ± 13 (men, 52 ± 13; women, 53 ± 12).

### Laboratory methods

Blood samples were obtained from an antecubital vein into vacutainer tubes containing EDTA in the morning after an overnight fasting period. Blood samples were centrifuged within 2 h at the survey site. Plasma was transferred to separate labeled tubes and transported immediately on dry ice at prearranged intervals to the Xinjiang Key Laboratory of Cardiovascular Disease. Serum concentrations of serum total cholesterol, triglycerides, low-density lipoprotein (LDL), high-density lipoprotein (HDL), and fasting glucose were measured by the Clinical Laboratory Department of the First Affiliated Hospital of Xinjiang Medical University with a biochemical analyzer (Dimension AR/AVL Clinical Chemistry System, Newark, NJ, USA) [22, 23].

### Blood pressure measurement

A mercury sphygmomanometer was used to measure blood pressure in the sitting position after a 10-min rest period. During the 30-min preceding measurement, the subjects were required to refrain from smoking or consuming caffeine. The appearance of the first sound was used to define systolic blood pressure, and the disappearance of sound was used to define diastolic blood pressure [24]. Two readings each of systolic and diastolic blood pressures were recorded, and the average of each measurement was used for data analysis. If the first two measurements differed by more than 5 mmHg, additional readings were taken.

### Definition of risk factors

Hypertension was defined as self-reported use of antihypertensive medication within the past 2 weeks or an average systolic blood pressure ≥140 mmHg, an average diastolic blood pressure ≥90 mmHg, or both. Diabetes was defined as fasting plasma glucose ≥7.0 mmol/L, use of insulin or oral hypoglycemic agents, or a self-reported history of diabetes. BMI ≥ 24 (kg/m^2^) were defined as overweight. Total cholesterol concentrations >6.22 mmol/L (240 mg/dl) were defined as hypercholesterolemia. Triglyceride concentrations >2.26 mmol/L (200 mg/dl) were defined as hypertriglyceridemia. LDL cholesterol concentrations >4.14 mmol/L (160 mg/dl) were defined as high LDL cholesterol. HDL cholesterol concentrations <1.04 mmol/L (40 mg/dl) were defined as low HDL cholesterol. Dyslipidemia was defined as TG ≥ 2.26 mmol/l, TC ≥ 6.22 mmol/l, LDL-C ≥ 4.14 mmol/l, HDL-C < 1.04 mmol/l, or if receiving a lipid-lowering drug [25].

### Statistical analysis

Statistical analysis was conducted using SPSS version 17.0 for Windows (SPSS Inc., Chicago, IL, USA). Continuous variables were expressed as sex-specific means and standard deviations, and discrete variables were expressed as sex-specific proportions. Analysis of variance was used for continuous variables, and the chi-square test was used for categorical variables. A value of *P* < 0.05 indicates a statistically significant difference. Age standardization was performed by the direct method by using the Han population according to the population census data of Xinjiang in 2000 as the standard population. The sensitivity and specificity of each TG/HDL level for the detection of hypertension, dyslipidemia, diabetes, and two or more of these risk factors were calculated by creating dichotomous variables for each TG/HDL value. Additionally, the distance on the receiver operating characteristic (ROC) curve of each TG/HDL value was calculated as the square root of [(1 − sensitivity)^2^ + (1 − specificity)^2^]. The TG/HDL value with the shortest distance on the ROC curve was considered in the determination of optimal cutoff. The overall performance of the TG/HDL test for detecting cardiovascular risk factors was assessed by computing the area under the curves (AUC). An AUC of 1 is considered to have perfect discriminatory power, and an AUC of 0.5 suggests that the discriminatory power is no better than chance.

## Results

As presented in Table 1, with the increase of TG/HDL-C, the serum total cholesterol increased from 4.13 ± 1.13 to 5.15 ± 1.10 (*P* < 0.001), the fasting glucose increased from 5.15 ± 1.68 to 6.05 ± 2.66 (*P* < 0.001), the HDL cholesterol decreased from 1.60 ± 0.50 to 0.93 ± 0.37 (*P* < 0.001), and the LDL cholesterol decreased from 3.07 ± 0.83 to 2.63 ± 0.94 (*P* < 0.001) in men. In Table 2, with the increase of TG/HDL-C, the serum total cholesterol increased from 4.22 ± 1.09 to 5.23 ± 1.24 (*P* < 0.001), the fasting glucose increased from 4.88 ± 1.12 to 5.77 ± 2.03 (*P* < 0.001), the HDL cholesterol decreased from 1.64 ± 0.60 to 0.85 ± 0.34 (*P* < 0.001), and the LDL cholesterol decreased from 3.06 ± 0.95 to 2.56 ± 0.88 (*P* < 0.001) in women. We also noticed the triglycerides increased from 0.85 ± 0.78 to 3.39 ± 2.27 (*P* < 0.001) in women with the increase of TG/HDL-C; we did not notice the same trend in men.

**Table 1:** Age-standardized cardiovascular disease risk factors in the Chinese Han men by TG/HDL-C category

	TG/HDL-C ≤ 0.5	0.5 < TG/HDL-C ≤1.0	1.0 < TG/HDL-C ≤1.5	1.5 < TG/HDL-C ≤2.0	2.0 < TG/HDL-C ≤2.5	TG/HDL-C > 2.5	*P* value
Men							
Population distribution (%)	460 (16.5 %)	639 (22.9 %)	536 (19.2 %)	398 (14.3 %)	253 (9.1 %)	502 (18.0 %)	
Age (years)	52.64 ± 13.20	53.99 ± 13,94	53.54 ± 13.96	51.71 ± 12.74	50.27 ± 12.30	49.44 ± 12.01	<0.001
Systolic blood pressure (mmHg)	133.19 ± 19.69	132.92 ± 19.35	133.91 ± 17.67	135.99 ± 17.93	134.60 ± 18.43	136.92 ± 18.56	<0.001
Diastolic blood pressure (mmHg)	86.00 ± 15.14	85.13 ± 14.80	86.86 ± 14.59	88.87 ± 15.19	87.89 ± 15.35	90.36 ± 15.39	<0.001
Total cholesterol (mmol/L)	4.13 ± 1.13	4.42 ± 0.99	4.68 ± 0.95	4.81 ± 0.93	4.92 ± 0.99	5.15 ± 1.10	<0.001
HDL cholesterol (mmol/L)	1.60 ± 0.50	1.39 ± 0.39	1.24 ± 0.36	1.15 ± 0.36	1.10 ± 0.36	0.93 ± 0.37	<0.001
LDL cholesterol (mmol/L)	3.07 ± 0.83	3.03 ± 0.91	2.83 ± 0.84	2.79 ± 0.81	2.76 ± 0.88	2.63 ± 0.94	<0.001
Triglycerides (mmol/L)	1.17 ± 1.47	1.02 ± 0.30	1.55 ± 0.49	1.99 ± 0.62	2.47 ± 0.83	3.95 ± 2.61	<0.001
Fasting glucose (mmol/L)	5.15 ± 1.68	5.23 ± 1.93	5.39 ± 1.68	5.45 ± 1.40	5.55 ± 1.70	6.05 ± 2.66	<0.001
BMI (kg/m^2^)	25.07 ± 3.21	24.97 ± 3.44	25.92 ± 3.09	26.08 ± 3.03	26.07 ± 2.81	26.63 ± 3.24	<0.001

*TG/HDL-C* triglycerides**/**high-density lipoprotein cholesterol, *HDL* high-density lipoprotein, *LDL* low-density lipoprotein, *BMI* body mass index

**Table 2:** Age-standardized cardiovascular disease risk factors in the Chinese Han women by TG/HDL-C category

	TG/HDL-C ≤ 0.5	0.5 < TG/HDL-C ≤1.0	1.0 < TG/HDL-C ≤1.5	1.5 < TG/HDL-C ≤2.0	2.0 < TG/HDL-C ≤2.5	TG/HDL-C > 2.5	*P* value
Women							
Population distribution (%)	607 (20.4 %)	988 (33.3 %)	520 (17.5 %)	358 (12.1 %)	220 (7.4 %)	276 (9.3 %)	
Age (years)	48.65 ± 11.51	51.34 ± 12.11	54.40 ± 11.99	55.81 ± 11.64	56.71 ± 11.90	56.34 ± 11.25	<0.001
Systolic blood pressure (mmHg)	123.97 ± 18.67	128.27 ± 20.33	133.87 ± 20.97	135.88 ± 20.42	139.69 ± 21.42	139.11 ± 20.68	<0.001
Diastolic blood pressure (mmHg)	78.96 ± 15.93	81.59 ± 15.08	83.79 ± 15.55	86.07 ± 15.52	84.52 ± 15.57	87.77 ± 15.16	<0.001
Total cholesterol (mmol/L)	4.22 ± 1.09	4.52 ± 0.96	4.88 ± 0.97	5.00 ± 0.99	5.05 ± 1.28	5.23 ± 1.24	<0.001
HDL cholesterol (mmol/L)	1.64 ± 0.60	1.36 ± 0.36	1.21 ± 0.33	1.08 ± 0.33	0.97 ± 0.31	0.85 ± 0.34	<0.001
LDL cholesterol (mmol/L)	3.06 ± 0.95	3.06 ± 0.95	2.73 ± 0.82	2.71 ± 0.89	2.66 ± 0.85	2.56 ± 0.88	<0.001
Triglycerides (mmol/L)	0.85 ± 0.78	0.99 ± 0.28	1.50 ± 0.42	1.86 ± 0.57	2.15 ± 0.68	3.39 ± 2.27	<0.001
Fasting glucose (mmol/L)	4.88 ± 1.12	5.02 ± 1.51	5.30 ± 1.46	5.46 ± 1.80	5.56 ± 1.88	5.77 ± 2.03	<0.001
BMI (kg/m^2^)	23.53 ± 3.26	24.00 ± 3.62	25.21 ± 3.77	25.22 ± 3.49	25.89 ± 3.39	25.86 ± 3.45	<0.001

*TG/HDL-C* triglycerides**/**high-density lipoprotein cholesterol, *HDL* high-density lipoprotein, *LDL* low-density lipoprotein, *BMI* body mass index

The prevalence of hypercholesterolemia increased from 16.7 to 44 % in men and from 15.7 to 49.3 % in women as the TG/HDL-C increased (both *P* < 0.001), the prevalence of low HDL cholesterol increased from 10.9 to 65.1 % in men and from 7.6 to 73.6 % in women as the TG/HDL-C increased (both *P* < 0.001), and the prevalence of diabetes and hypertriglyceridemia showed no trend as TG/HDL-C increased for both men and women. The prevalence of hypertension increased from 21.6 to 55 % in women as the TG/HDL-C ratio increased (*P* < 0.001); however, we did not observe any trend in the prevalence of hypertension as TG/HDL-C increased for men (Tables 3 and 4).

**Table 3:** Age-standardized prevalence of risk factors in the Chinese Han men by TG/HDL-C category

	TG/HDL-C ≤ 0.5 (%)	0.5 < TG/HDL-C ≤1.0 (%)	1.0 < TG/HDL-C ≤1.5 (%)	1.5 < TG/HDL-C ≤2 (%)	2.0 < TG/HDL-C ≤2.5 (%)	TG/HDL-C > 2.5 (%)	*P* value
Men							
Hypertension	34.60	37.80	42.20	46.40	35.50	47.00	<0.001
Diabetes	4.90	5.50	9.30	9.00	8.30	17.10	<0.001
Hypercholesterolemia	16.70	17.40	28.00	32.20	36.80	44.00	<0.001
High LDL cholesterol	48.00	42.90	36.60	35.30	34.00	28.30	<0.001
Low HDL cholesterol	10.90	12.80	31.70	43.70	50.60	65.10	<0.001
Hypertriglyceridemia	15.30	3.30	29.70	63.10	82.60	94.40	<0.001

*TG/HDL-C* triglycerides**/**high-density lipoprotein cholesterol

Hypertension was defined as SBP ≥ 140 mmHg and/or DBP ≥ 90 mmHg and/or current antihypertensive drug use. Diabetes was defined as fasting plasma glucose ≥7.0 mmol/L and/or current diabetes drug use. Hypercholesterolemia was defined as TC ≥ 6.22 mmol/L, high LDL cholesterol was defined as LDL-C ≥ 4.14 mmol/L, low HDL cholesterol was defined as HDL-C < 1.04 mmol/L, and hypertriglyceridemia was defined as TG ≥ 2.26 mmol/L

**Table 4:** Age-standardized prevalence of risk factors in the Chinese Han women by TG/HDL-C category

	TG/HDL-C ≤ 0.5 (%)	0.5 < TG/HDL-C ≤1.0 (%)	1.0 < TG/HDL-C ≤1.5 (%)	1.5 < TG/HDL-C ≤2 (%)	2.0 < TG/HDL-C ≤2.5 (%)	TG/HDL-C > 2.5 (%)	*P* value
Women							
Hypertension	21.60	32.00	41.80	48.30	51.40	55.00	<0.001
Diabetes	3.20	4.70	8.30	9.80	8.60	13.80	<0.001
Hypercholesterolemia	15.70	21.40	36.00	40.50	45.00	49.30	<0.001
High LDL cholesterol	44.30	43.40	28.50	28.50	30.60	25.40	<0.001
Low HDL cholesterol	7.60	15.10	35.00	52.20	62.30	73.60	<0.001
Hypertriglyceridemia	7.40	1.50	26.30	54.70	66.80	87.70	<0.001

*TG/HDL-C* triglycerides**/**high-density lipoprotein cholesterol

Hypertension was defined as SBP ≥ 140 mmHg and/or DBP ≥ 90 mmHg and/or current antihypertensive drug use. Diabetes was defined as fasting plasma glucose ≥7.0 mmol/L and/or current diabetes drug use. Hypercholesterolemia was defined as TC ≥ 6.22 mmol/L, high LDL cholesterol was defined as LDL-C ≥ 4.14 mmol/L, low HDL cholesterol was defined as HDL-C < 1.04 mmol/L, and hypertriglyceridemia was defined as TG ≥ 2.26 mmol/L

The population distribution of each TG/HDL-C ratio and the sensitivity, specificity, and distance on the ROC curve for the detection of hypertension, dyslipidemia, diabetes, and ≥2 of these risk factors for men and women were shown in Tables 5 and 6, respectively. In men, the cutoff for dyslipidemia and hypertension was 1.3. The shortest distances on the ROC curve of diabetes and ≥2 of these risk factors were the same. A TG/HDL-C ratio of 1.3 appeared to be the optimal TG/HDL-C ratio cutoff in men. In women, the shortest distance on the ROC curve for diabetes and dyslipidemia was 1.0. The shortest distance on the ROC curve for hypertension was 0.9, and ≥2 of these risk factors was 1.1. A TG/HDL-C ratio of 1.0 appeared to be the optimal TG/HDL-C ratio cutoff in women.

**Table 5:** Sensitivity (Sens), specificity (Spec), and distance in the receiver operating characteristic (ROC) curvefor TG/HDL-C cutoffs in the Chinese Han men

		Hypertension			Dyslipidemia			Diabetes			≥2 risk factors		
TG/HDL-C cutoffs	Percentile	Sens	Spec	Distance in ROC curve	Sens	Spec	Distance in ROC curve	Sens	Spec	Distance in ROC curve	Sens	Spec	Distance in ROC curve
Men		%			%			%			%		
0.5	9.1	0.89	0.15	0.85	0.98	0.24	0.76	0.92	0.15	0.86	0.97	0.16	0.84
0.6	13.8	0.85	0.22	0.79	0.96	0.33	0.67	0.88	0.20	0.81	0.95	0.23	0.77
0.7	18.3	0.80	0.30	0.73	0.94	0.44	0.57	0.85	0.27	0.75	0.94	0.30	0.70
0.8	24.0	0.76	0.35	0.69	0.92	0.52	0.49	0.81	0.32	0.71	0.91	0.36	0.65
0.9	28.1	0.71	0.39	0.67	0.90	0.59	0.42	0.77	0.37	0.67	0.89	0.41	0.60
1.0	32.1	0.67	0.44	0.65	0.88	0.66	0.37	0.74	0.42	0.64	0.86	0.46	0.56
1.1	36.6	0.63	0.49	0.64	0.85	0.72	0.32	0.70	0.46	0.62	0.83	0.51	0.52
1.2	40.0	0.59	0.52	0.63	0.82	0.77	0.29	0.66	0.50	0.61	0.80	0.55	0.49
1.3	43.9	0.55	0.56	*0.63*	0.79	0.82	*0.28*	0.62	0.54	0.60	0.77	0.60	0.47
1.4	47.5	0.50	0.60	0.64	0.74	0.85	0.30	0.57	0.58	*0.60*	0.71	0.64	*0.46*
1.5	51.4	0.46	0.64	0.65	0.69	0.88	0.34	0.53	0.62	0.60	0.67	0.67	0.47
1.6	55.1	0.42	0.67	0.67	0.64	0.91	0.37	0.49	0.66	0.61	0.63	0.70	0.48
1.7	57.9	0.39	0.69	0.68	0.61	0.92	0.40	0.47	0.68	0.62	0.59	0.73	0.49
1.8	60.8	0.35	0.72	0.71	0.56	0.95	0.44	0.43	0.72	0.64	0.54	0.76	0.52
1.9	63.6	0.32	0.73	0.73	0.53	0.96	0.48	0.40	0.74	0.66	0.50	0.78	0.55
2.0	65.5	0.31	0.74	0.74	0.50	0.96	0.50	0.38	0.75	0.66	0.48	0.79	0.56

The cutoff for dyslipidemia and hypertension was 1.3 and for diabetes and ≥2 risk factors was 1.4

**Table 6:** Sensitivity (Sens), specificity (Spec), and distance in the receiver operating characteristic (ROC) curve for TG/HDL-C cutoff in the Chinese Han women

		Hypertension			Dyslipidemia			Diabetes			≥2 risk factors		
TG/HDL-C cutoffs	Percentile	Sens	Spec	Distance in ROC curve	Sens	Spec	Distance in ROC curve	Sens	Spec	Distance in ROC curve	Sens	Spec	Distance in ROC curve
Women	%	%			%			%			%		
0.5	14.5	0.85	0.26	0.75	0.94	0.30	0.70	0.89	0.22	0.79	0.97	0.25	0.75
0.6	21.8	0.79	0.36	0.68	0.90	0.41	0.60	0.84	0.31	0.71	0.92	0.34	0.67
0.7	29.6	0.72	0.44	0.62	0.86	0.51	0.51	0.78	0.39	0.65	0.88	0.43	0.59
0.8	37.0	0.65	0.54	0.58	0.82	0.62	0.42	0.72	0.47	0.60	0.84	0.52	0.51
0.9	43.3	0.60	0.59	*0.58*	0.79	0.69	0.38	0.67	0.52	0.58	0.82	0.58	0.46
1.0	47.6	0.55	0.64	0.58	0.76	0.74	*0.35*	0.63	0.57	*0.57*	0.77	0.63	0.44
1.1	51.7	0.51	0.68	0.59	0.71	0.78	0.37	0.58	0.62	0.57	0.74	0.67	*0.42*
1.2	55.1	0.47	0.71	0.61	0.67	0.81	0.38	0.54	0.65	0.58	0.70	0.71	0.42
1.3	59.0	0.43	0.74	0.63	0.63	0.84	0.41	0.49	0.68	0.60	0.65	0.74	0.44
1.4	62.0	0.39	0.77	0.65	0.58	0.87	0.44	0.46	0.71	0.62	0.61	0.77	0.46
1.5	65.3	0.36	0.80	0.68	0.53	0.89	0.48	0.42	0.75	0.64	0.56	0.80	0.48
1.6	68.1	0.32	0.82	0.70	0.49	0.91	0.52	0.39	0.78	0.65	0.52	0.82	0.51
1.7	70.9	0.29	0.84	0.73	0.45	0.92	0.56	0.35	0.80	0.68	0.49	0.85	0.54
1.8	73.4	0.26	0.86	0.75	0.40	0.94	0.60	0.32	0.83	0.70	0.44	0.87	0.57
1.9	75.9	0.23	0.90	0.77	0.37	0.95	0.64	0.29	0.85	0.73	0.40	0.89	0.61
2.0	77.5	0.22	0.88	0.79	0.35	0.95	0.65	0.28	0.85	0.73	0.39	0.89	0.62

The cutoff for dyslipidemia and diabetes was 1.0, for hypertension was 0.9, and for ≥2 risk factors was 1.1

**Fig. 1:**
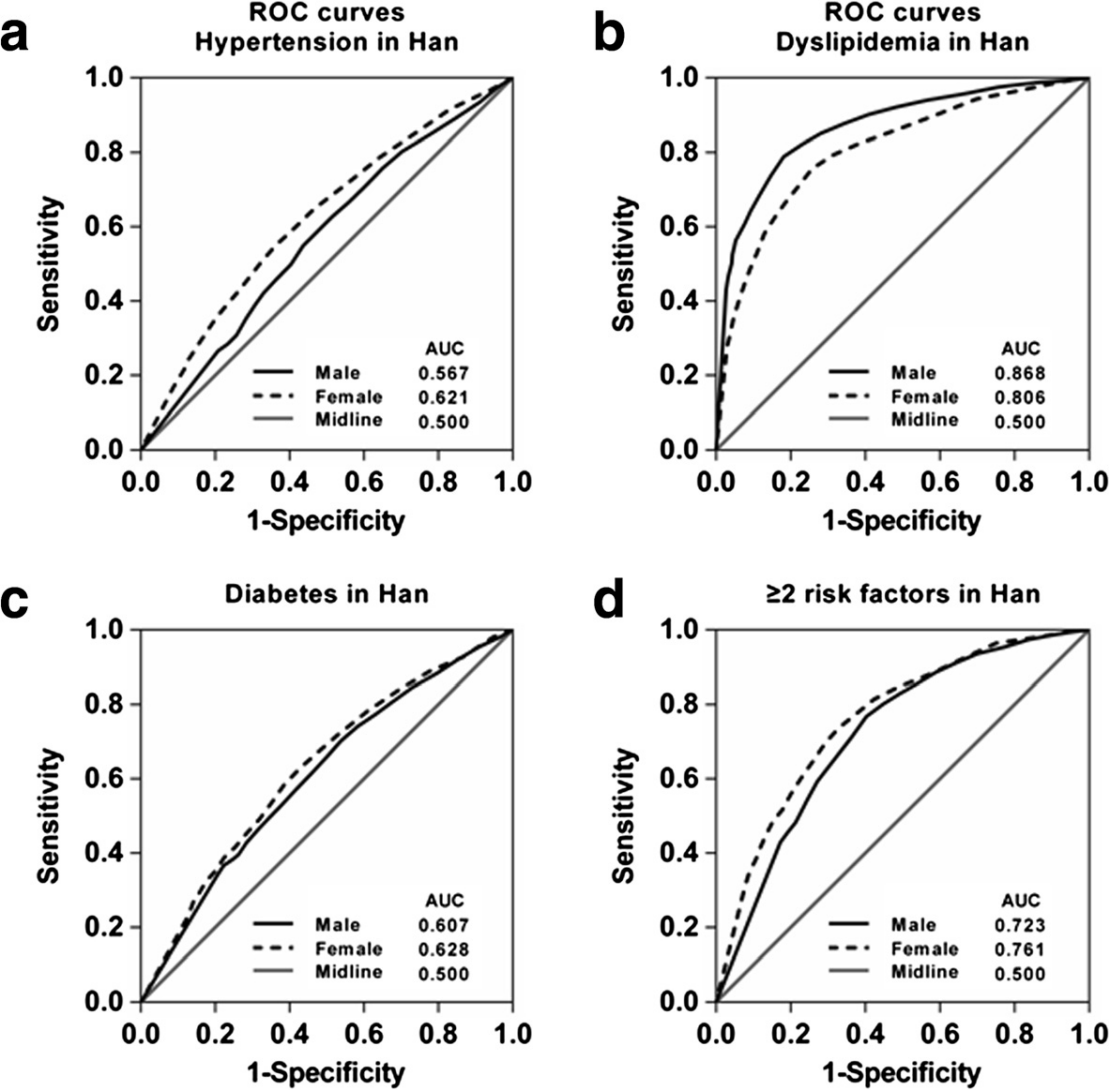
ROC curves to detect CVD risk factors by sex. **a** ROC curves for both men and women for the detection of hypertension, **b** dyslipidemia, **c** diabetes, and **d** ≥2 of these risk factors

The ROC curves for men and women were shown in Fig. 1. We found that the discriminatory power of TG/HDL-C ratio for cardiovascular risk factors was slightly better in women than in men.

## Discussion

Dyslipidemia is an important risk factor of coronary heart disease (CVD). Although some lipid variables were associated with the extent of coronary disease, the ratio of triglycerides to HDL cholesterol (TG/HDL-C) showed the strongest association with extent [26]. This study provides population-based data on the optimal cutoff of the triglyceride to HDL cholesterol  ratio for detecting cardiovascular risk factors in Chinese Han population in Xinjiang aged over 35 years. To our knowledge, this is the first large-scale, population-based, cross-sectional survey to estimate the TG/HDL-C ratio cutoff in the Han population in Xinjiang, China. We also found a relationship between the TG/HDL-C ratio and cardiovascular risk factors.

In addition, the TG to HDL-C ratio (TG/HDL-C) has been proposed as a useful lipid parameter associated with metabolic syndrome (MetS), insulin resistance (IR), and cardio-metabolic risk [15, 17, 26]. It has been successfully used in predicting the development of diabetes, coronary heart disease, cardiovascular events, and all-cause mortality [27–29]. MetS is a cluster of metabolic abnormalities characterized as central obesity, a raised level of triglycerides (TG), reduced high-density lipoprotein cholesterol (HDL-C), raised blood pressure (BP), and raised fasting plasma glucose (FPG) or previously diagnosed type 2 diabetes; all these factors are associated with cardiovascular disease [18]. TG/HDL-C is a relatively new atherogenic parameter for MetS, and the reports on this in the Chinese population are limited. According to the ATPIII and JIS criteria, the optimal cutoff for TG/HDL-C was 1.6 and 1.2 in men and 1.1 and 1.1 in women, respectively. The cutoff values of the markers to detect the MetS in Guangdong women is 0.88 [30] and 0.84 in Zhuhai women [31]; the cutoff value in Ghanaian women is 0.61 [32]. In Canada, men and women is 1.62 and 1.18, respectively [33]. Few studies of this cutoff value have focused on CVD in Han adults. In the present study, based on the sensitivity, specificity, and ROC calculations, the optimal cutoffs of TG/HDL-C ratio for Han men and women in Xinjiang were 1.3 and 1.0, respectively. Additionally, this study showed an increasing trend in the prevalence of hypertension, hypercholesterolemia, hypertriglyceridemia, and diabetes with higher TG/HDL-C among Han adults. These elevated cardiovascular risks are associated with MetS and IR. Current studies have reported that the cutoffs of BMI, waist circumference (WC), and waist-to-hip ratio (WHR) in Xinjiang were slightly higher comparing to the WHO criteria [33, 34]. Similarly, the cutoffs of TG/HDL-C ratio in Xinjiang are slightly higher than other regions and criterions.

The reasons why there are higher optimal cutoffs of TG/HDL among Han adults in Xinjiang are unclear. Possible reasons may be associated with differences in diet, living conditions, climate, and some important genes that regulate body fat distribution. First, the main reason may be a difference in diet in Xinjiang Han adults compared with Guangdong, Zhuhai, and Ghanaian. The Han population in Xinjiang consumes more meat, milk products, salt, and pasta than Asian populations in other regions. Second, a characteristic feature of the living conditions and climate in Xinjiang is that the temperature difference during day and night is considerable, summer is extremely hot, and winter is extremely cold. Therefore, drinking strong wine, eating more animal fat, and a higher salt intake in the Han population may lead to fat deposition thicker than other populations for adaptation to the external environment in Xinjiang. Third, certain genes that regulate body fat distribution may be different in different populations, so that body fat is more likely to accumulate [35–37]. In addition, the difference in optimal values of TG/HDL-C ratio between populations might be due to varied body size, physical activity, and metabolic status.

We also found that the TG/HDL-C cutoff in Han women in Xinjiang was the same as the ATPIII and JIS criteria, but it was higher than in Guangdong and Ghanaian. The reason why there are higher optimal cutoffs may be associated with differences of eating habits and lifestyles. We also found that the cutoffs in Han men were higher than women in Xinjiang, similar to the ATPIII and JIS criteria. The reason why there are higher cutoffs may be more smoking and alcohol consumption and less exercise.

The ratio TG/HDL-C, initially proposed by Gaziano et al., is an atherogenic index that has proven to be a highly significant independent predictor of cardiovascular disease, even stronger than TC/HDL-C and LDL-C/HDL-C [14]. The Copenhagen Male Study showed that TG/HDL-C levels can more accurately detect coronary disease [38]. The possible mechanism may be that high triglycerides and low HDL-C may lead to the accumulation of small and dense LDL-C, and these LDL-C particles may cause HDL-C particles to undergo accelerated catabolism, which could close the atherogenic circle [39, 40]. The current study indicates that the cutoff of TG/HDL-C ratio is a good predictor of CAD [41]. This specific ratio has the best sensitivity and specificity, and it will be an easy, non-invasive means of predicting the presence and extent of coronary atherosclerosis.

### Strengths and limitations

Our study has several strengths. It is the first representative sample of the general adult Han population in Xinjiang. These results may be generalized to the full adult population of Hans aged above 35 years. Additionally, we provided information on cutoffs and AUC for many anthropometric and atherogenic parameters stratified by sex. Future studies can use the cutoffs suggested here for the screening and intervention of cardiovascular disease in a representative sample of the adult Han population in Xinjiang. The limitation of this study is this is a cross-sectional study, and it may not able to predict the prognosis of the disorders. Future studies can use the ratio of TG/HDL cutoffs suggested here as a reference to evaluate the associated risk factors and intervention effects of dyslipidemia in other adult Han population.

## Conclusions

In conclusion, this study showed a TG/HDL-C ratio value of 1.3 and 1.0 in men and women, respectively, as an appropriate cutoff to distinguish high cardiovascular risk patients. The continuous relationship between cardiovascular disease risk factors and triglyceride/high-density lipoprotein cholesterol (TG/HDL-C) ratio was documented here. For all the anthropometric and atherogenic parameters, the ratio of TG/HDL-C cutoff had the best predicting ability of CVD in both men and women.
